# Prolines in linkers of multi-domain proteins: Intrinsic regulators of misfolding and aggregation

**DOI:** 10.1371/journal.pcbi.1014588

**Published:** 2026-07-31

**Authors:** Simona Manasra, Yaakov Levy

**Affiliations:** Department of Chemical and Structural Biology, Weizmann Institute of Science, Rehovot, Israel; University of Massachusetts Amherst, UNITED STATES OF AMERICA

## Abstract

Proline is a chemically unique amino acid that influences protein structure and slows peptide-bond formation during translation. While its enrichment in intrinsically disordered regions is well known, the functional importance of its organization along protein sequences remains unclear. Here, we performed a proteome-wide analysis of inter-domain linkers in human proteins to investigate how proline organization relates to domain architecture and folding demands. We find that clustered and consecutive prolines are enriched in linkers following topologically complex domains that require the formation of long-range contacts. This suggests that proline clusters may act as intrinsic sequence-encoded pauses during translation, helping coordinate co-translational folding. We further show that linkers with enriched proline clusters frequently flank aggregation-prone domains and that proteins containing such linkers tend to have longer cellular half-lives. Together, our results demonstrate that the organization of proline residues, beyond their overall abundance, is an important determinant of protein folding, aggregation, and stability, revealing a general mechanism by which amino acid sequences regulate protein behavior.

## Author summary

Proteins often consist of multiple domains connected by flexible linker regions. These linkers are typically disordered and are enriched in the amino acid proline, which has unusual structural and biochemical properties. While proline is known to influence protein structure and slow down protein synthesis when it appears in clusters, it remains unclear how its organization within sequences contributes to protein folding and stability. Here, we systematically analyzed inter-domain linkers across the human proteome to understand how proline arrangement relates to protein structure and function. We found that clusters of prolines are enriched in linkers that follow structurally complex domains, which require the formation of long-range contacts during folding. This suggests that proline clusters may act as intrinsic “pauses” during protein synthesis, allowing these domains more time to fold properly. In addition, we observed that linkers with enriched conserved proline clusters frequently occur next to aggregation-prone domains, indicating a potential role in reducing misfolding and aggregation. Together, our results reveal that the organization of proline residues, beyond their overall abundance, is an important sequence-encoded feature that helps coordinate protein folding and maintain protein stability. This work highlights how subtle patterns in amino acid sequences can regulate fundamental biological processes at the proteome scale.

## Introduction

Proline is unusual among amino acids because its side chain covalently bonds to the backbone nitrogen, restricting torsional freedom and eliminating the backbone amide hydrogen. These constraints propagate through multiple levels of protein organization. At the secondary structure level, proline frequently introduces kinks, caps α-helices, and interrupts β-strands, and more broadly promotes intrinsic disorder by disfavoring stable secondary-structure formation. In intrinsically disordered regions (IDRs), the presence of proline alters compaction by reducing hydrogen bonding capacity while restricting conformational sampling. Proline can also act as a structural barrier to limit β-sheet propagation. Molecular dynamics simulations of prion proteins have shown that β-sheet elongation is prevented by proline residues flanking the sheet, whereas substitution of these prolines with valine permitted further extension [1]. In folded proteins, proline-rich segments create hinges that modulate domain movements. Beyond structure, proline-rich motifs serve as recognition sites for modular binding domains such as SH3, WW, and EVH1, linking them to signaling function [2].

### Proline organization along the sequence affects intrinsically disordered regions conformations

Proline not only widely occurs in IDRs but also affects their conformations: clustered prolines (i.e., prolines with no more than two intervening non-proline residues in sequence) result in expanded peptide conformations whereas isolated prolines impose compacted conformations via the formation of backbone turns [3]. The abundance of clustered prolines in the IDRs of the human proteome increases with the sequence length when the percentage of isolated prolines remain constant, suggesting that longer IDRs may favor more extended and flexible conformations, achieved through specific proline patterns. Moreover, aromatic–proline interactions can stabilize cis conformers and nucleate compact states, reducing conformational entropy and favoring the formation of structural hubs within disordered regions [4]. The conformation of IDRs is known to be functionally important in many contexts, for example in modulating the spacing and dynamics between protein domains [5] or in regulating accessibility to proteases. IDRs are frequently targeted by proteases and can determine protein lifetime by serving as initiation sites for proteasomal degradation [6]. If proline distribution can indeed shift IDR conformational ensembles, then it may provide a sequence-encoded mechanism to tune these processes. In this way, proline organization in the sequence has the potential to act not only as a local structural modifier but also as a regulator of broader protein behaviors such as degradation, folding coordination, and inter-domain communication.

### Proline clusters change translation dynamics

The unusual geometry of proline is also influential during protein synthesis, where it can affect the translation. Several experimental studies have shown that proline can act as a strong initiator of translation stalling when they cluster together. Proline cyclic structure eliminates the backbone amine and slows peptide bond formation at the P-site. Polyproline stretches destabilize the peptidyl-tRNA and adopt geometries incompatible with the ribosome exit tunnel, leading to strong stalling [7]. Cells overcome this challenge by employing EF-P in bacteria and eIF5A/aIF5A in eukaryotes and archaea, which rescue stalled ribosomes by stabilizing peptide bond formation. Experimental studies show that proline-rich motifs, such as XPPX, can trigger stalls whose efficiency depends on the sequence context [8], with measured pausing ranging from about 8–70% of ribosomes arrested in E. coli in vitro depending on the upstream residue [9] and stalling events persisting for several minutes under in vitro translation conditions [10].

Even subtle alterations in translation dynamics can have profound effects on how efficiently and accurately a polypeptide attains its native structure. Translation inefficiency disrupts proper folding, increasing the likelihood of misfolding and aggregation [11].

### Proline positioning may affect co-translational folding efficiency as well as intrinsically disordered region–related processes

There is increasing evidence that slowing or stalling of translation is important in the context of co-translational folding. During co-translational folding a nascent polypeptide chain begins folding as it emerges from the ribosome what may reduce kinetic competition with non-native states. By modulating the rate at which the nascent chain emerges from the ribosome, cells can synchronize the appearance of folding units with the time needed for them to acquire their structure. Slower elongation at specific sites reduces competition with non-native states, while excessively rapid elongation can expose large intermediates that are prone to misfolding and aggregation. As detailed in [12], the ribosome modulates folding through both mechanical confinement in its exit tunnel and kinetic control of translation elongation. Faster translation rates can lead to premature emergence of entire domains before upstream regions fold properly, while slower translation at key positions (e.g., domain boundaries or hydrophobic segments) enables domains to fold with minimal interference. In fact, folding intermediates are often structured to fold efficiently at specific chain lengths, and the timing of their synthesis, connected to the rate at which the ribosome moves along the mRNA, can dramatically influence folding yield [13,14].

The fact that co-translational folding can guide proper structure implies that folding is shaped not only by external factors, like chaperones, but also by the mRNA sequence itself, as it determines the speed and efficiency of translation. This effect is not limited by the amino acids differences: synonymous codons, which encode the same amino acid, can have vastly different impacts on elongation rate. Non-optimal codons, which are decoded by low-abundance tRNAs, can slow down translation and, in some cases, trigger ribosome collisions that affect initiation rates or lead to degradation signals [15]. Since ribosome interacts with several codons at a time, non-optimal codon clusters are supposed to increase this effect. Although relatively few codon-specific regulatory mechanisms have been experimentally explored, increasing evidence shows that clusters of non-optimal codons play a functional role, determining the translation speed [16]. For example, non-optimal codons were found to locate mostly downstream the protein tertiary structures in *E*. *coli*, playing a functional role in folding optimization [17].

A similar principle may apply to amino acid encoded pauses: just as codon bias modulates translation kinetics, clusters of prolines can create sequence-driven stalls that fine-tune the timing of folding. Bioinformatic analyses in *E. coli* reveal that despite their cost, polyproline motifs are enriched near start sites, inter-domain boundaries, and transmembrane helices, suggesting that such stalling is selectively maintained to coordinate folding and insertion [18]. This pattern suggests that translation stalling caused by polyproline motifs is selectively preserved to support efficient co-translational folding and membrane insertion. Yet, such regions outside the domains are disordered and therefore generally proline-rich, limiting interpretability. While experimental studies have established that clustered prolines cause ribosome stalling, it remains unclear when this stalling serves a beneficial function. The open question is whether stalling is functionally required for specific structural contexts, such as domains with particular folding challenges, rather than being a byproduct of sequence composition. If proline clusters act not only as structural modifiers in IDRs but also as intrinsic regulators of translation dynamics, then their presence may connect sequence composition directly to folding of multi-domain proteins.

In this study, we investigate whether the organization of prolines, particularly clustered prolines, serves as a sequence-encoded mechanism to regulate folding and stability in multi-domain proteins. Specifically, we examine how proline-dependent modulation of disordered-region conformations relates to processes such as aggregation avoidance, and whether clusters of prolines positioned near domain boundaries may influence co-translational folding through their impact on translation dynamics. To address these questions, we perform a proteome-wide analysis of human multi-domain proteins, quantifying the distribution, organization, and evolutionary conservation of prolines within inter-domain linkers and relating these patterns to structural properties of neighboring domains, including topological complexity and aggregation propensity, as well as broader protein-level characteristics.

## Materials and methods

### Dataset of the proteins, domains and linkers

The human reference proteome (UniProt ID: UP000005640), that contains 7705 multi-domain proteins, was used as the primary dataset. For comparison, selected analyses were also performed on the *Escherichia coli* reference proteome (UniProt ID: UP000000625) with 1324 proteins. In addition, a subset of 704 multi-domain mouse proteins with experimentally measured turnover rates was taken from the study of Rolfs et al. (2021) [19]. Protein domain boundaries were annotated using HMMER [20] search against the Pfam-A database [21]. Because linker regions were defined operationally as non-domain intervals between Pfam-annotated domains, some unannotated structured regions, coiled-coil regions, functional low-complexity regions may be included in this category. Overlapping domains in HHMER results were merged into unified domain regions whenever their annotated residue ranges overlapped, we did not apply an additional overlap threshold. This was done to avoid introducing artificial short linker segments caused by minor boundary differences between neighboring hits. In addition, regions annotated as repeats in the Pfam database were identified and merged with adjacent similar repeat domains, forming extended repeat units. This merging was performed based on the fact that individual repeat units are typically short and often unable to fold independently and are thus structurally and functionally interdependent. Proteins containing only a single annotated domain were excluded from the analysis. Linkers were defined as the amino acid sequences separating two non-overlapping domain regions, at least 3 amino acids long. Each analyzed segment was grouped as a domain(i) –linker–domain(i + 1) triplet, so protein with *n* domains has *n – 1* triplets. Proteins with GO [22] annotations indicating binding to domains known to interact with polyproline motifs, such as SH3, SH2, actin, talin, integrin, vinculin, Arp2/3, profilin, receptor tyrosine kinase, protein kinase C, phosphotyrosine, PTB, PDZ, EH domain, lysine-acetylated histone, and dynein Pham domains were filtered out, as these interactions could bias the analysis. The final dataset consists of 15892 triplets from human proteins and 1919 triplets from *E. Coli* proteins. Protein structures were modeled with AlphaFold2 [23], protein domains structures were fetched from the full AlphaFold structures. To assess confidence at the domain level, we calculated the mean pLDDT for each extracted domain and used this measure to define a high-confidence subset of domains with mean pLDDT values above 70.

### Proline abundance and clustering significance

**Fig 1:**
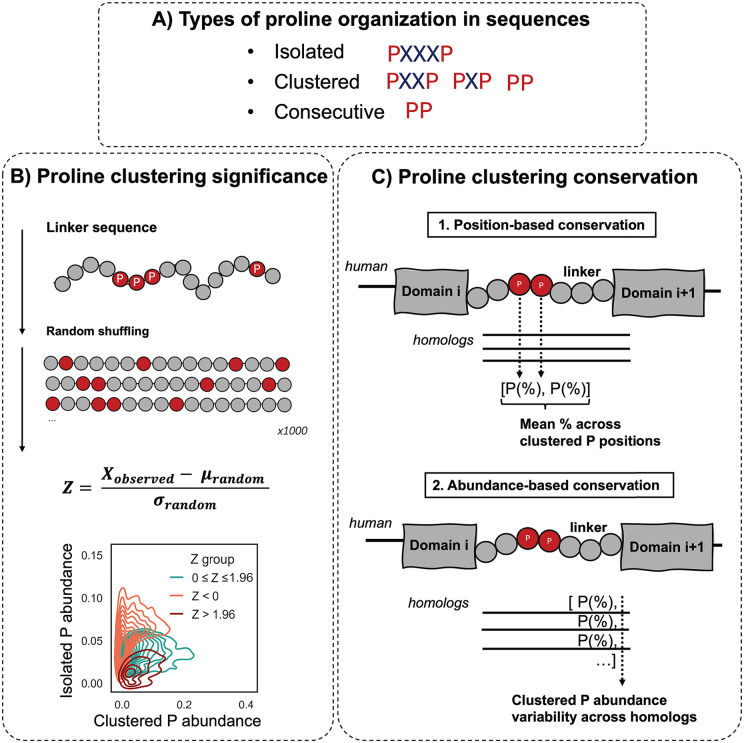
Clustered proline significance and conservation. **(A)** Definition of proline organization patterns. **(B)** Clustered proline significance was estimated using random shuffling of linker sequences and Z-score calculation to compare observed versus expected clustering. The resulting plot shows clustered and isolated proline abundances grouped by Z-score: negative values indicate dispersed, isolated prolines; intermediate values reflect mixed distributions; and high positive values denote significant enrichment in clustered prolines. **(C)** Schematic overview of two complementary approaches to proline-conservation analysis. The linker region between adjacent domains is highlighted in pink. (1) Position-based conservation: for each proline within a human cluster, the proportion of homologs retaining a proline at the corresponding alignment position is calculated, and the mean is derived across clustered proline positions. (2) Abundance-based conservation: for each homolog, the percentage of clustered prolines within the linker is determined, followed by coefficient of variation estimation across homologs.


Prolines in the linker regions were classified in three categories: consecutive, clustered and isolated. Consecutive prolines are the motifs containing two or more prolines located directly next to each other in the sequence. Proline clusters are the motifs with prolines separated with no more than two non-proline amino acid residues; these also includes consecutive prolines. Isolated prolines don’t have other proline residues within two residues upstream or downstream in the sequence (Fig 1A). The abundance of prolines in each category was calculated as the number of prolines in this category divided by the length of the linker region. A Z-score was calculated for each linker to evaluate whether the observed abundance of clustered prolines exceeded random expectation. For each sequence, the clustered proline abundance was compared to a null distribution generated by random shuffling of the same linker sequence while preserving amino acid composition. The mean and standard deviation of clustered proline abundances from the null distribution were used to compute the Z-score as

$$\begin{array}{c} Z=\;\frac{X_{observed}-\;\mu_{random}\;}{\sigma_{random}}, \end{array}$$

where X_observed_ is the observed abundance of clustered prolines in the linker, μ_random_ - the mean of the null distribution (mean abundance after random shuffling of the linker sequence), and σ_random_ - its standard deviation.

Negative Z-scores indicate lower clustering than expected by chance, Z-scores around zero indicate no enrichment, and positive Z-scores indicate higher-than-expected clustering. Z-scores above 1.96 correspond to a significance threshold of p < 0.05 (two-tailed), indicating enrichment in clustered prolines beyond random expectation. Linkers with negative Z-scores (Z < 0) show very low clustered proline abundance, but often retain a high fraction of isolated prolines, indicating that prolines are present but dispersed. Linkers with intermediate Z-scores (0 ≤ Z ≤ 1.96) tend to display modest clustered abundance alongside isolated residues, consistent with random-like distributions. By contrast, linkers with significant enrichment (Z > 1.96) show a clear shift toward higher clustered proline abundance and almost no isolated prolines, highlighting that in these cases prolines strongly segregate into clusters rather than remaining dispersed (Fig 1B). In short linkers (< 50 aa), the Z-score distribution is skewed, making Z > 1.96 essentially unattainable: because high proline percentages are easily achieved by chance in such short sequences, non-random enrichment can only be estimated for longer linkers.

### Clustered proline conservation

For each protein containing at least one linker with clustered prolines, a multiple sequence alignment was generated using its homologous sequences. Homologs of the reference human proteins were identified using MMseqs2 [24] by searching against the UniRef50 database [25]. To ensure a meaningful set of homologous proteins, an e-value cutoff of 1e-4 and a maximum cutoff of 1000 sequences were used. Then only sequences within ±20% of the reference protein length were retained. The homolog sequences were aligned with Clustal Omega, which employs hidden Markov models to generate accurate alignments [26]. To maintain structural comparability, for each linker only the homologs with the similar domain lengths within ±20% of the reference domains were included in the final alignment. Two different approaches were used to estimate the evolutionary conservation of prolines across species: (1) conservation of individual prolines at specific positions within clusters in the reference human protein, and (2) conservation of clustered proline abundance across species (Fig 1C). In the first approach, the conservation of each clustered proline was calculated as the percentage of homologs that retained a proline at the corresponding alignment position. For each linker, the mean conservation across all clustered prolines was computed. In the second approach, the abundance of clustered prolines was calculated for each homolog, and coefficient of variation (CV) of these values, defined as the standard deviation relative to the mean, was computed across species.

Phylogenetic trees were constructed from multiple sequence alignments of full-length proteins containing the target linker regions. For each reference human protein, a distance matrix based on sequence identity was calculated, and a neighbor-joining tree was generated using Biopython Bio.Phylo package [27]. To assess whether high clustered proline abundance is phylogenetically conserved or randomly distributed among homologs, we quantified the evolutionary “clustering” of this trait using the Fitch parsimony score [28]. For each linker and its corresponding set of homologous proteins, tips in the phylogenetic tree were labeled as either “high” or “low” based on whether their clustered proline abundance exceeded the 10th percentile of nonzero values within that linker. The minimum number of transitions needed to explain the observed distribution across the tree, known as the Fitch parsimony score, was then computed using a manual implementation of the Fitch algorithm. To enable comparison across proteins and linkers with varying numbers of homologs, the parsimony score was normalized by dividing by the total number of homologs in the tree.

### Estimating folding complexity with contact order

One of the approaches to estimate protein folding rates computationally is based on the concept of Contact Order [29]. Absolute contact order (CO) measures the average sequence separation between residue pairs that are in contact in the 3D structure, reflecting the topological complexity of folding. Its normalized form, Relative Contact Order (RCO), accounts for protein length. While CO gives insight into the absolute sequence distances required for residue contacts, RCO captures the topological complexity of the fold. This becomes particularly informative in the context of co-translational folding, where the protein folds progressively as it’s being synthesized. Contact order and relative contact order were measured for all the domains of the dataset using their AlphaFold predicted models. Residue-residue contacts were defined as pairs of residues whose Cα atoms are within 6 Å of each other. For each domain, the sequence separation between contacting residues was computed, and CO and RCO were defined as


$$\begin{array}{c} CO=\;\frac{\text{1}}{N}\sum_{\left(a_{i},a_{j}\right)}L_{ij},\;RCO=\;\frac{\text{1}}{N*L}\sum_{\left(a_{i},a_{j}\right)}L_{ij}, \end{array}$$

where L_ij_ is the sequence separation between contacting residues i and j, N is the total number of residue-residue contacts, L is the length of the domain in residues.

CO and RCO measures are used as topological descriptors that also serve as a basic scale for understanding domain organization. Both measures are closely related to domain length and secondary structure composition: for example, the highest RCO values generally correspond to small β-rich domains. These domains cannot have high absolute contact order due to the small length. β-sheets generally exhibit higher contact order due to inter-sheet connections (S1 Fig).

### Aggregation prediction

The estimation of domain aggregation was done by using three different tools, integrating different methods. TANGO is a sequence-based algorithm that predicts β-sheet aggregation-prone regions in unfolded polypeptide chains using principles of statistical mechanics [30]. ANuPP is a machine learning–based predictor that classifies hexapeptides as aggregation-prone based on learned features, showing high accuracy in identifying nucleating regions [31]. Aggrescan3D offers a structure-based approach, incorporating solvent exposure and conformational dynamics to evaluate aggregation-prone patches on protein surfaces. It includes a comprehensive aggregation score database based on AlphaFold2-predicted models [32]. Scores from these tools were Min-Max scaled and averaged to obtain a unified domain aggregation score.

## Results

### Prolines distribution across protein regions

**Fig 2:**
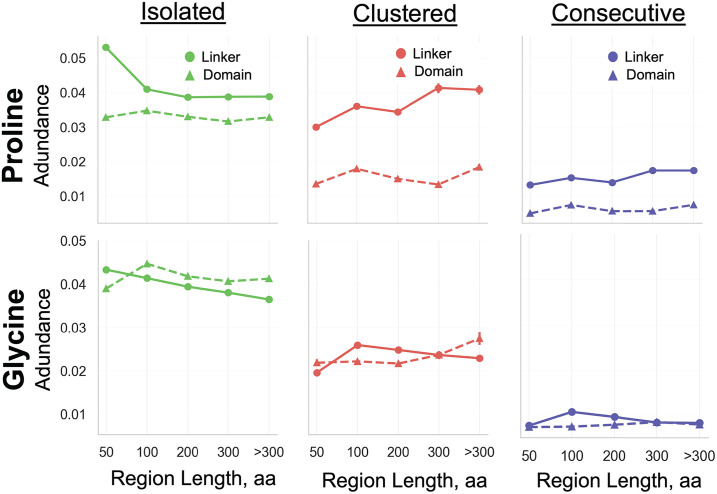
Abundance of proline and glycine patterns across different protein regions. Solid lines correspond to linker regions, and dashed lines correspond to domains.

To compare how disorder-promoting residues, such as proline and glycine, distribute across protein regions, we examined their abundance of in domains and linkers as a function of region length (Fig 2). Both residues are generally known to be enriched in intrinsically disordered regions (IDRs), yet they display different patterns when analyzed as consecutive (i.e., no spacing between the residues), clustered (i.e., spacing of up to two residues), or isolated groups (i.e., spacing greater than two residues) (see Methods section for definition of these sequence organization). Proline preferentially accumulates in linkers: clustered and consecutive prolines are markedly more common in linkers than in domains, consistent with their role in disrupting secondary structure and introducing local flexibility. Glycine, in contrast, is distributed more evenly between linkers and domains. A notable trend emerges in long linkers, where clustered prolines steadily increase in abundance and eventually surpass isolated prolines. This indicates that prolines are not randomly dispersed but instead tend to form clusters in long IDRs, what was also shown in similar analysis before [3]. Although linkers are not exclusively disordered and may incorporate structured segments such as α-helices, the consistently higher percentage of clustered and consecutive prolines compared to the corresponding glycine motifs strongly suggests that proline plays a more specific organizational role.

### Clustered proline enrichment increases with structural complexity

**Fig 3:**
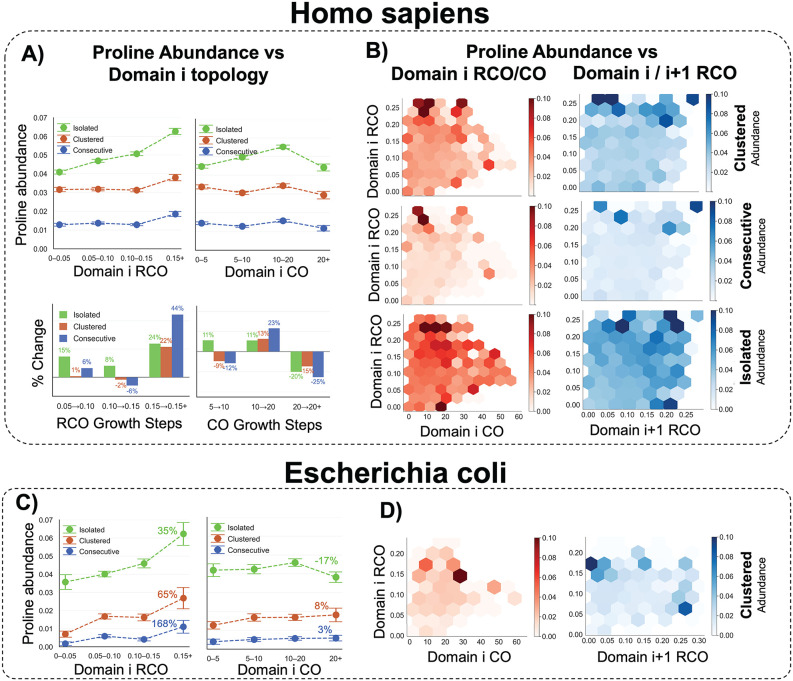
Relationship between domain i topology and proline clustering in downstream linkers. **(A-B)** Homo sapiens: Abundance of isolated, clustered, and consecutive prolines in downstream linkers as a function of upstream domain Relative Contact Order (RCO, left) or Contact Order (CO, middle). The histogram points the number of analyzed linkers in each group. Right: heatmap plot showing combined effects of domain RCO and CO on consecutive proline abundance in linkers. **(C-D)** Escherichia coli: Equivalent analysis in the bacterial proteome.

If prolines in linker regions facilitate co-translational folding by inducing ribosome stalling, their abundance should vary with the structural complexity of the preceding domain. We analyzed proline abundance in downstream linkers according to the structural complexity of the preceding domains, measured by their absolute contact order (CO) and relative contact order (RCO). Both metrics are linked to folding kinetics: higher values indicate slower folding due to the requirement to establish long-range intramolecular contacts. In the human proteome, proline abundance in downstream linkers increases markedly with RCO (Fig 3A, left), statistical comparisons across groups of increasing structural complexity confirmed significant differences in proline organization (one-way ANOVA test). The effects were observed for isolated (p = 5.53 × 10 ⁻ ³⁰ clustered (p = 2.21 × 10 ⁻ ⁶), and consecutive (p = 4.35 × 10 ⁻ ⁷) prolines. Follow-up pairwise comparisons between group means (Tukey’s HSD test) showed that linkers following the most topologically complex domains (RCO > 0.15) differ significantly from all other groups for clustered prolines (vs 0–0.05: p = 1.5 × 10 ⁻ ⁵, 95% CI [0.0043, 0.0147]; vs 0.05–0.10: p = 9.2 × 10 ⁻ ⁵, 95% CI [0.0027, 0.0105]; vs 0.10–0.15: p = 1.6 × 10 ⁻ ⁴, 95% CI [0.0025, 0.0104]) and consecutive prolines (vs 0–0.05: p = 4.4 × 10 ⁻ ⁶, 95% CI [0.0033, 0.0102]; vs 0.05–0.10: p = 3.3 × 10 ⁻ ⁵, 95% CI [0.0020, 0.0073]; vs 0.10–0.15: p = 4.8 × 10 ⁻ ⁵, 95% CI [0.0020, 0.0073]), whereas isolated prolines increase more gradually across bins. By contrast, no clear pattern emerged when domains were grouped by CO as captured by RCO (Fig 3A, right). The strongest effect of domain complexity is observed for consecutive prolines, which increase by more than 40% in linkers downstream of high-RCO domains. Unlike RCO, CO showed no consistent relationship with proline clustering in downstream linkers: clustered proline enrichment was observed specifically after high-RCO domains, even when their CO values differed (Fig 3B, left). Taken together, these findings suggest that proline clustering in linkers is specifically tied to the complexity of establishing a domain’s three-dimensional fold during synthesis. Notably, the upstream domain i shows the stronger association with proline clustering, whereas the downstream domain i + 1 alone does not produce a comparable effect in human proteins (Fig 3B, right). A similar enrichment patterns were observed in the simpler *E. coli* proteome (Fig 3C-3D), although overall clustered proline levels were lower. As a robustness check for the contact order estimation, we repeated the analysis using an atom-based contact definition with an 8 Å cutoff and obtained the same qualitative outcome; this analysis is provided in the Supplementary Information (S2 Fig).

We next extended the analysis to all 20 amino acids to test whether the observed behavior was specific to proline (S3 and S4 Fig). Although glutamate and serine displayed broadly similar clustering trends in linker regions, likely reflecting their compatibility with flexible and intrinsically disordered sequence environments, the strong association with the upstream rather than downstream flanking domain was observed specifically for proline. This suggests that general linker enrichment can be separated from the more specific domain-context dependence identified for proline.

**Fig 4:**
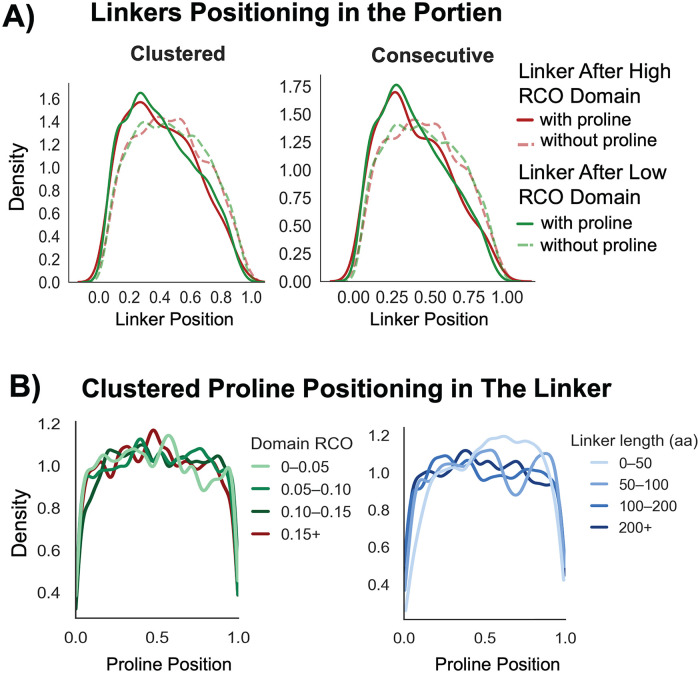
Positional distribution of linkers and clustered prolines. **(A)** Kernel density of the positions of linkers within proteins that contain clustered (left) and consecutive prolines (right), comparing high- vs. low-RCO upstream domains. **(B)** Density of clustered proline positions within linkers across different domain i RCO ranges (left) and linker lengths(left), showing uniform internal distributions.

We next examined whether clustered proline-enriched linkers (that have at least one clustered proline motif) preferentially occur at specific positions within proteins, and whether this distribution is influenced by domain complexity. Using kernel density estimates of linker start positions normalized by protein length, we observed a strong enrichment of clusters-containing linkers closer to N-terminal regions. This difference between the groups was highly significant for both clustered (Mann-Whitney U test: p = 3.11 × 10 ⁻ ⁴⁸, Cliff’s δ = -0.145) and consecutive proline-containing linkers (p = 1.14 × 10 ⁻ ⁴⁷, Cliff’s δ = -0.165), with the positional bias being more pronounced for consecutive motifs. However, there was no significant difference between high- and low-RCO domains, indicating that while domain complexity promotes greater overall clustering, it does not influence where these linkers are positioned within proteins (Fig 4A). We used RCO > 0.15 as a cutoff to define a high-RCO subset. In our dataset, this threshold lies above the 80th percentile of the empirical RCO distribution and therefore captures a relatively small upper-tail fraction of domains with comparatively high topological complexity. Since linker-position KDEs can be influenced by multidomain architecture, particularly because proteins with fewer domains contribute disproportionately to early linker positions, we repeated the analysis after stratifying proteins by domain count (2 domains, 3–4 domains, and 5 + domains). This showed that the positional shift is strongest in proteins with fewer domains, but it remains detectable across domain-count groups, especially for linkers containing consecutive prolines (S5 Fig).

When focusing on the internal distribution within the linker itself, we found that clustered and consecutive prolines occupy similar positions regardless of RCO or linker lengths, with broad distributions across the linker sequence (Fig 4B). Thus, domain complexity drives the overall abundance of proline clustering but does not strongly bias where inside the linker these motifs appear.

### Linker length shapes the relationship between domain complexity and proline clustering

**Fig 5:**
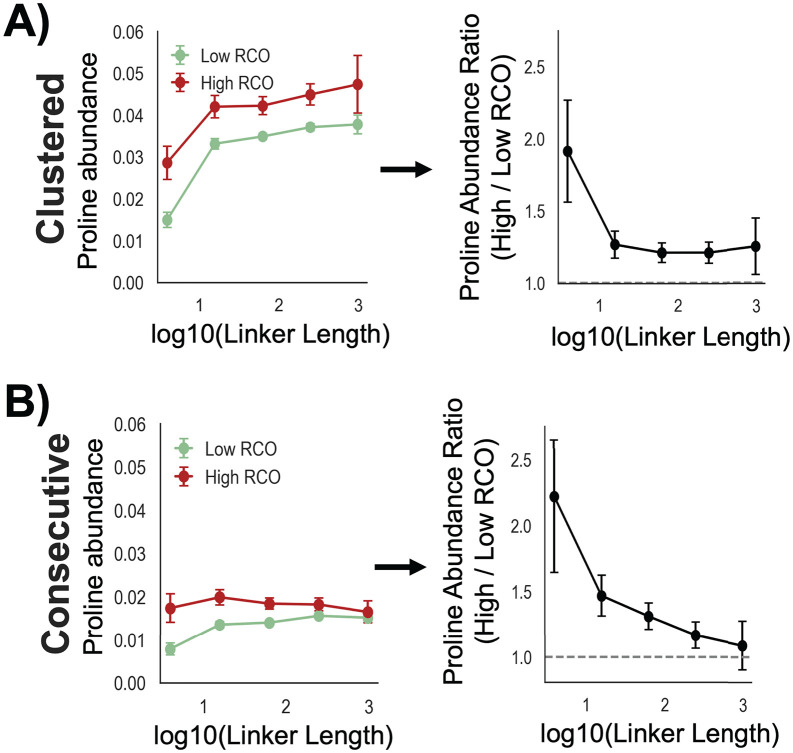
Linker length modulates the effect of domain complexity on proline enrichment. Line plots (left) display proline abundance across linker length bins, comparing high- and low-RCO groups. Difference plots (right) highlight that the enrichment of clustered (A) and especially consecutive (B) prolines is strongest in short linkers, with the effect diminishing as linker length increases.

Because linker length directly influences translation time, and therefore the interval between the translation of two adjacent domains, we next examined whether linker length affects the relationship between domain complexity and clustered proline enrichment in the linker. We grouped domains into two categories: those with extremely high RCO (>0.15) and those with lower complexity (RCO < 0.15) and analyzed how proline pattern differs between these two categories across different linker lengths (Fig 5). The largest differences in clustered proline enrichment between high- and low-RCO groups were observed in short linkers, especially for consecutive prolines. In these short linkers, the abundance of consecutive prolines was approximately two-fold greater downstream of highly complex domains compared to the rest. This difference gradually decreased as linker length increased, becoming negligible for longer linkers. Because short linkers are translated rapidly, enhanced consecutive prolines may serve as critical translational brakes to allow nascent domains more time to fold before the next domain emerges.

### Clustered prolines show evolutionary conservation patterns

**Fig 6:**
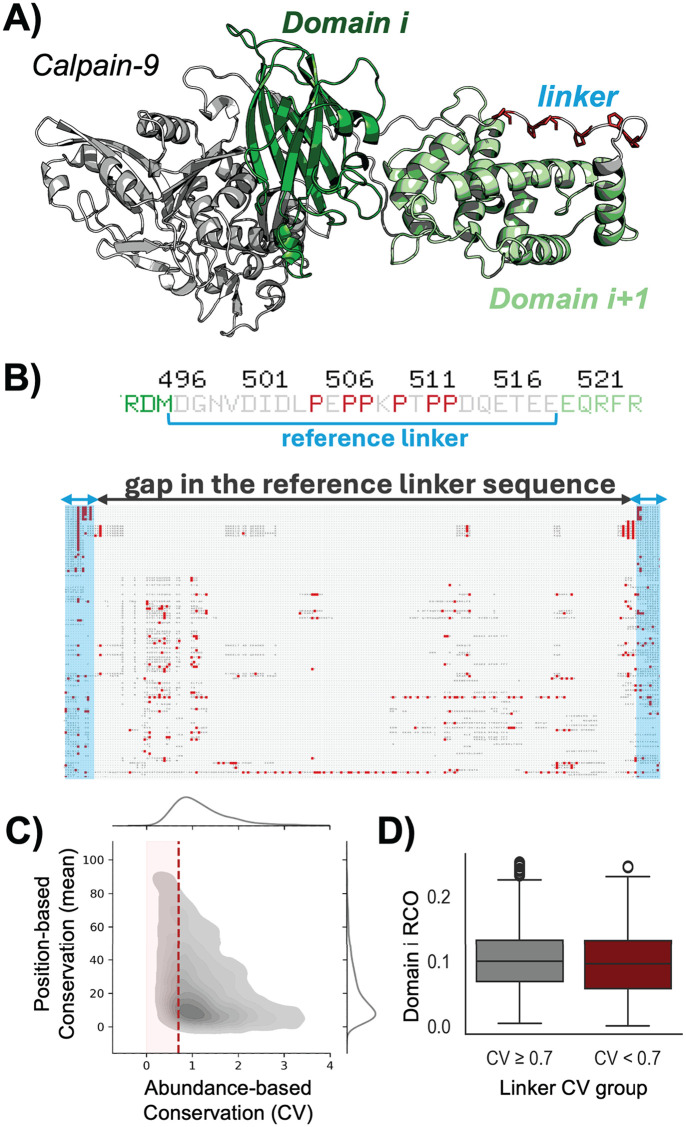
Conservation of clustered proline abundances. **(A)** Structure of the Calpain-9 protein (O14815), with highlighted domain(i) –linker–domain(i + 1) triplet. **(B)** Multiple sequence alignment of the linker from Calpain-9 protein, with prolines colored in red. Regions in blue refer to the sequences aligned with the reference human protein. **(C)** Joint distribution of position-based mean conservation and abundance-based conservation of the full dataset. **(D)** Boxplot of domain i relative contact order (RCO) grouped by abundance-based conservation of the following linker, measured as the coefficient of variation (CV) of clustered-proline abundance across homologs.

To assess the evolutionary conservation of clustered prolines within linkers, we employed two measures: position-based conservation, which quantifies residue conservation at each alignment position, and abundance-based conservation, expressed as the coefficient of variation (CV) of clustered proline abundance across homologs. Given the functional role of clustered prolines in translation stalling, abundance-based conservation may be more meaningful, as the presence of prolines, rather than their exact positions, better captures biologically relevant signals. Indeed, in some cases we observe strong conservation of abundance even when the positional pattern varies, such as when clusters occur in different linker regions. These cases still yield low CV values because the overall abundance is similar between homologs, a signal that may be even more informative than strict positional conservation. One such example is Calpain-9 protein, which contains homologs with clustered prolines positioned in distinct parts of the linker, including regions that correspond to gaps in the reference sequence (Fig 6A-6B). The consistent presence across the alignment results in a low CV despite positional variation, reinforcing the idea that abundance-based conservation can capture meaningful evolutionary signals that positional metrics may overlook (Fig 6C). However, this conservation of abundance does not appear to be restricted to proteins with specific structural characteristics: there is no clear dependence on RCO (Fig 6D).

**Fig 7:**
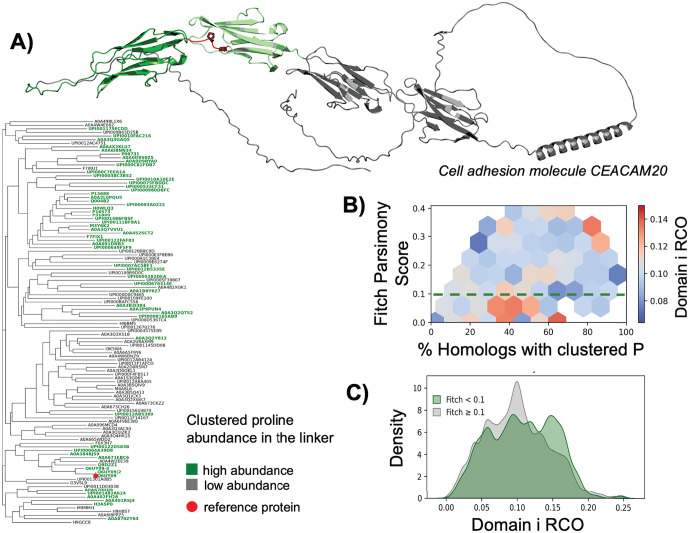
Lineage patterns in clustered proline abundances. **(A)** Structure of the Cell adhesion molecule CEACAM20 (Q6UY09) with clustered prolines highlighted in red; domain i is shown in dark green and domain i + 1 in pale green. The phylogenetic tree of Q6UY09 homologs is shown on the left, with homologs containing clustered prolines in the linker highlighted in green. **(B)** Normalized Fitch parsimony score versus the percentage of homologs containing clustered prolines, colored by the mean RCO of domain **i.**(**C**) Density plot of domain i RCO, separated by low and high Fitch score of the following linker.

Even when high abundance is not shared across all homologs, a meaningful evolutionary footprint can still emerge when presence/absence patterns are examined in a phylogenetic context. To illustrate how clustered proline enrichment can be distributed across homologs, in the protein Cell adhesion molecule CEACAM20 (Q6UY09) the linker region shows some lineage-specific patterns (Fig 7A). Homologs can be classified into two categories based on which linkers show high clustered proline abundance in a binary way. When mapped onto the phylogenetic tree, these categories form clades, rather than a random mix, suggesting that enrichment in the given linker is conserved within lineages. In the large-scale analysis, this principle was applied to each linker independently. For each linker, we calculated the percentage of homologs exceeding the threshold and compared this to the normalized Fitch parsimony score, which indicates whether homologs containing clustered prolines are phylogenetically grouped or dispersed across the tree. A Fitch score of ~0.1 or less generally indicates a phylogenetic conservation, indicating few inferred state changes across the tree, but such low values can occur trivially when the motif is present in around 0 or 100% of homologs. In contrast, linkers following high-RCO domains often achieve similarly low Fitch scores even at intermediate motif frequencies (~30–70%) indicating that the presence/absence pattern is not random but clustered within specific clades (Fig 7B). Linkers with low Fitch scores (<0.1), indicating phylogenetic conservation, display a distinct peak at higher domain RCO values (Fig 7C). These findings point to lineage-specific conservation: the clustered proline high abundance is not universally present, nor sporadically scattered, but rather retained within certain evolutionary branches with few inferred gain/loss events.

### Proline clustering emerges in aggregation contexts

**Fig 8:**
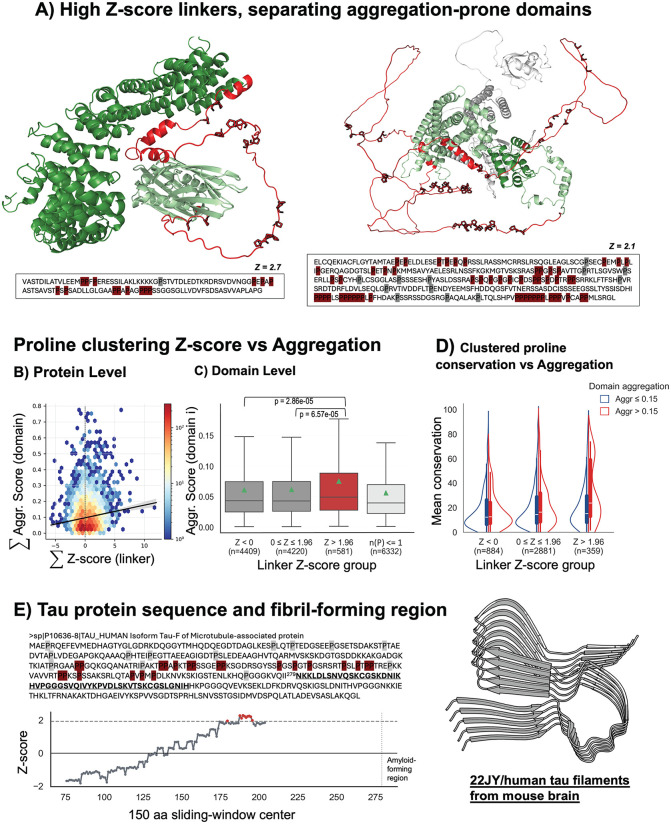
Z-score based significance of proline clustering. **(A)** Representative proteins (AP-2 complex subunit alpha-2 on the left and Delphilin on the right) with proline-rich linkers in red. Domain i is shown in dark green, domain i + 1 in pale green. Within linker sequences, clustered prolines are marked in red and isolated ones in gray. **(B)** Scatter plot of cumulative linkers Z-scores per protein versus its domains aggregation scores. **(C)** Aggregation scores for domain i, grouped by linker Z-score ranges. **(D)** Mean position-based conservation of clustered prolines in the linker, grouped by linker Z-score ranges and domain i RCO. **(E)** Human Tau sequence illustrating a proline-rich region upstream of the fibril-forming segment. Clustered proline residues are highlighted in red, the aggregation-prone fibril-forming region is underlined. The proline clustering Z-score, calculated using a 150-residue sliding window across the disordered region upstream of the fibril-forming segment, gradually increases and reaches the threshold (1.96, see Fig 1) near the fibril-forming segment.

To quantify whether clustered proline enrichment is greater than expected by chance, we calculated a Z-score for each linker by comparing the observed number of clustered prolines to a null model based on random sequence shuffling. For short linkers, Z-scores are not informative because even a small number of prolines are more likely to appear “clustered” after shuffling. Considering the overall abundance of prolines, non-random clustering is difficult to reproduce under random shuffling in long linkers. Indeed, in high Z-score linkers many prolines are concentrated into clusters rather than scattered individually, as illustrated in representative protein linkers (Fig 8A). At the protein level, proteins with higher cumulative Z-scores across their linkers also tend to display higher aggregation propensity in their domains (Fig 8B). The aggregation score represents a normalized composite index combining multiple aggregation predictors per domain. As these predictors are intercorrelated, the score integrates their shared variance to yield a unified domain-level aggregation measure. At the domain level, significantly clustered linkers are more frequently adjacent to domains with elevated aggregation scores (Fig 8C). For upstream domains (domain i), statistical comparisons of group medians (Mann–Whitney U test) show that those flanking linkers with Z > 1.96 have markedly higher aggregation scores compared to both Z < 0 (p = 2.86e-05) and 0 ≤ Z ≤ 1.96 (p = 6.57e-05). For downstream domains (domain i + 1), the effect is weaker but still significant, with Z > 1.96 higher than Z < 0 (p = 8.18e-03) and 0 ≤ Z ≤ 1.96 (p = 5.18e-03). This suggests that proline clustering is not a random feature but arises in contexts where domains on either side of the linker are intrinsically more aggregation prone. Such positioning suggests a potential role for clustered prolines in buffering aggregation risk during folding. We next asked whether these clusters are also preserved in homologous proteins at the same positions. Consistently, this most aggregation-prone group also shows higher positional conservation of clustered prolines only within the non-random clustering (Z > 1.96), suggesting that their placement in linkers is maintained more strictly across homologs (Fig 8D). Together, these observations indicate an association between non-random proline clustering and domains with higher predicted aggregation propensity. This pattern is particularly evident in long linkers, where the precise positioning of clusters may reflect functional or structural constraints. As an illustrative biological example, we examined human Tau, a well-known aggregation-prone protein that contains an extended proline-rich region adjacent to its fibril-forming sequence. Tau represents a somewhat different case from the multidomain proteins analyzed above, because it is largely intrinsically disordered and lacks a canonical domain-linker architecture. Nevertheless, it provides an informative example of a proline-rich sequence region positioned immediately upstream of an aggregation-prone segment located toward the C-terminus The proline-clustering Z-score increases progressively across the 150-residue region preceding the fibril-forming segment, reaching the significance threshold (Z = 1.96; see Fig 1) near the fibril-forming segment (Fig 8E).

## Discussion

The unusual chemical features may endow proline with multiple molecular roles. It has been proposed to modulate translation speed by inducing ribosome pausing, to create rigid hinges or sharp turns that shape tertiary geometry, to influence the compactness of intrinsically disordered regions, and to provide recognition motifs for proline-binding modules. While each of these functions has been proposed or observed in specific contexts, their relative importance within multi-domain proteins remains to be clarified. Here we focused on full-length proteins, where inter-domain linkers provide natural laboratories for sequence-driven regulation. In this study, we show that clustered prolines are strongly enriched in inter-domain linkers. Compared with isolated prolines or with proline occurrence inside domains, clustered and consecutive motifs accumulate preferentially in linkers, and their abundance rises with linker length until clusters surpass isolated prolines. We found that proline clusters are more common in linker regions following domains with high topological complexity, as measured by relative contact order (RCO). However, this pattern was not observed when domains were grouped by absolute contact order (CO), highlighting the specific importance of RCO in this context. These findings suggest that polyproline stretches may help coordinate co-translational folding by slowing down translation near slow-folding domains. By giving these complex regions more time to fold before the next domain begins to emerge, proline-induced stalling could improve folding efficiency and reduce misfolding. This highlights a potential sequence-level mechanism that links local translation dynamics to structural demands during protein synthesis. Our results are consistent with earlier work [17], which showed that rare codons are enriched near domains boundaries to slow translation and support proper folding. We show that the amino acid sequence itself can play a similar role and that the specific positioning of clustered prolines after particular domains is an important factor. While codon bias adjusts translation speed only subtly, from roughly 5–3 codons per second, what corresponds to about 0.2 to 0.3 seconds per codon [33], polyproline motifs introduce much stronger pauses, natural “breaks” in the translational rhythm, lasting several seconds [34], far exceeding typical codon-related fluctuations.

Though some studies have questioned the use of RCO as a predictor of folding rate, we suggest that during co-translational folding, it is not just folding rate but the timing of when folding begins and ends that matters. Since translation and folding happen simultaneously, long domains naturally gain more time to fold as their sequence emerges from the ribosome. In contrast, short domains with high topological complexity, (high RCO domains), must but cannot fold quickly before the next region is translated. This timing perspective also explains why short linkers exhibit the clearest difference in proline clustering. Translation time of the linker is correlated with its length - shorter linkers are translated faster than longer ones, and even a modest enrichment of consecutive prolines can create a meaningful pause, providing extra time for the upstream complex domain to reach a stable fold. The effect is weaker in longer linkers, where translation itself already provides a longer window. From an evolutionary perspective, since the functional impact of proline in this context is not tightly tied to its exact position within a linker, it is inherently harder to estimate and to compare across homologs than classical positional conservation. Still, we find examples in which clustered prolines are well conserved in overall abundance even when their precise positions differ among homologs. This conservation of abundance, rather than exact position, suggests that maintaining a certain level of proline-mediated effects can be important even when local sequence arrangements vary. However, such conserved abundance is not accompanied by systematic differences in structural properties, such as contact order, suggesting that direct structural constraints are limited. Interestingly, homologs that share high clustered-proline abundance tend to group together on the phylogenetic tree, particularly in proteins where the preceding domains have high RCO. This pattern hints that the tendency to maintain clustered prolines may have evolved alongside the folding topology of these complex domains, even though the signal is not strong enough to represent a universal rule. At the same time, interpreting proline conservation remains challenging because proline serves multiple, context-dependent roles, and its accumulation may arise from distinct selective pressures across proteins rather than a single unifying cause. We propose that proline-rich motifs function as local, sequence-level regulators of co-translational folding, complementing broader evolutionary strategies that tune domain topology and folding order to the kinetics of translation. Such a mechanism is also reflected in evolutionary analysis of two-domain proteins, which shows that N-terminal domains are systematically shorter and predicted to fold faster than their C-terminal counterparts, and this arrangement thought to facilitate co-translational folding and reduce misfolding risk during synthesis [35].

For longer linkers, we observe a different but complementary pattern. When these linkers contain proline clusters whose enrichment is unlikely to arise by chance, they are frequently flanked by aggregation-prone domains. The basis of this association remains unclear. One possibility is that proline-rich linkers may influence the local sequence environment of aggregation-prone domains, for example by affecting folding kinetics or by increasing spatial separation between neighboring domains. Consistent with a potential functional relevance, clustered prolines in these linkers also show higher positional conservation, indicating that their placement within the linker may be subject to stronger evolutionary constraints. Such conservation suggests that proline clustering could contribute to linker properties that influence domain organization or inter-domain communication. This suggests that proline clustering might serve both kinetic and structural protective roles, depending on the context of the flanking domains and the time available for co-translational folding.

Proline’s impact on intrinsically disordered regions extends far beyond its role in shaping inter-domain linkers. IDRs are central to many biological processes, and their behavior is exquisitely sensitive to sequence-encoded conformational biases. As an example of such a broader effect, we compared experimentally measured protein half-lives in the mouse proteome [19]. We found out that proteins with long half-lives were not only longer on average but also tended to contain linkers with higher clustered proline abundance (S6 Fig). Because proteasomal degradation often initiates within IDRs, the localized conformational constraints imposed by clustered prolines could reduce the flexibility or accessibility required for efficient proteasomal engagement and unfolding. Although the turnover dataset is limited in size, this relationship suggests that proline organization may influence proteostasis. However, with this analysis alone, it remains unclear whether this reflects a genuine function or simply a by-product of the presence of clustered prolines.

These observations also underscore questions that remain open. Because proline is both chemically distinctive and biophysically influential, its impact on protein architecture likely extends beyond what we have measured here. Proline’s ability to alter local flexibility, backbone geometry, and translation kinetics means that small changes in its distribution could propagate to large-scale effects on domain packing, inter-domain communication, and the spatial organization of multi-domain proteins. Addressing these questions will be essential for fully understanding how proline patterns shape protein structure and function.

## Supporting information

S1 Fig Distribution of domain topologies in contact order space. Relative Contact Order (RCO) is plotted against Contact Order (CO), with the background heatmap colored by (top) domain length and (bottom) β-sheet content. Example domains are highlighted: a β-sandwich domain (A6NHM9, residues 193–315; length 122 aa; CO = 25.23, RCO = 0.207), an ankyrin repeat (A0A0A6YYL3, residues 104–330; length 226 aa; CO = 8.64, RCO = 0.038), and a transmembrane domain (A0AV02, residues 44–415; length 371 aa; CO = 39.26, RCO = 0.106). The secondary structure composition of the domains was calculated using the DSSP algorithm. The score represents the percentage of residues classified as β-sheets among all structured residues.
(PDF)

S2 Fig Proline organization across domain CO/RCO bins using 6 Å and 8 Å contact definitions. Proline abundance in downstream linkers is shown for isolated, clustered, and consecutive proline motifs after stratifying upstream adjacent domains by RCO or CO. The analysis was repeated using contact definitions based on C-alpha distances of 6 Å and 8 Å.
(PDF)

S3 Fig Linker and domain abundance patterns of 20 amino acids across region lengths. For each amino acid, proline abundance in linker regions and annotated domains is shown across bins of region length. Amino-acid organization is presented separately for isolated, clustered, and consecutive proline, allowing comparison of linker-specific enrichment with the corresponding domain background across different region sizes.
(PDF)

S4 Fig Clustered proline abundance of all 20 amino acids across different RCO adjacent domains. Hexbin plots show the abundance of each clustered amino acid in linkers positioned between domain i and domain i + 1, plotted as a function of the domains RCO. Color intensity indicates the mean clustered abundance. This analysis was used to show that the asymmetric dependence on flanking-domain topology is specific to proline.
(PDF)

S5 Fig Positional distribution of proline-containing linkers across protein domain-count groups. Kernel density estimates show linker start positions normalized by protein length for linkers containing clustered or consecutive prolines. Proteins were stratified into 2-domain, 3–4-domain, and 5 + -domain groups to control for potential bias introduced by overall domain number. The positional shift toward more N-terminal locations remains detectable across groups, with the strongest effect observed in proteins containing fewer domains.
(PDF)

S6 Fig Mouse proteome experimental data for protein half-life. (A) Abundance of proline motifs across protein linkers analysis shows patterns consistent with the human data. (B) Proteins half-lifes distribution. (C) Boxplots comparing proteins with short (≤15 h) and long (>15 h) half-lives. Left: protein length. Right: maximum clustered proline abundance across linkers within each protein.
(PDF)
