# Supplementary material for: Prolines in linkers of multi-domain proteins: Intrinsic regulators of misfolding and aggregation
Source: PLoS Comput Biol. 2026 Jul 31;22(7):e1014588. doi: 10.1371/journal.pcbi.1014588 (PMC13456264; doi:10.1371/journal.pcbi.1014588)
Supplement: Supplementary file 3 [file pcbi.1014588.s003.pdf]

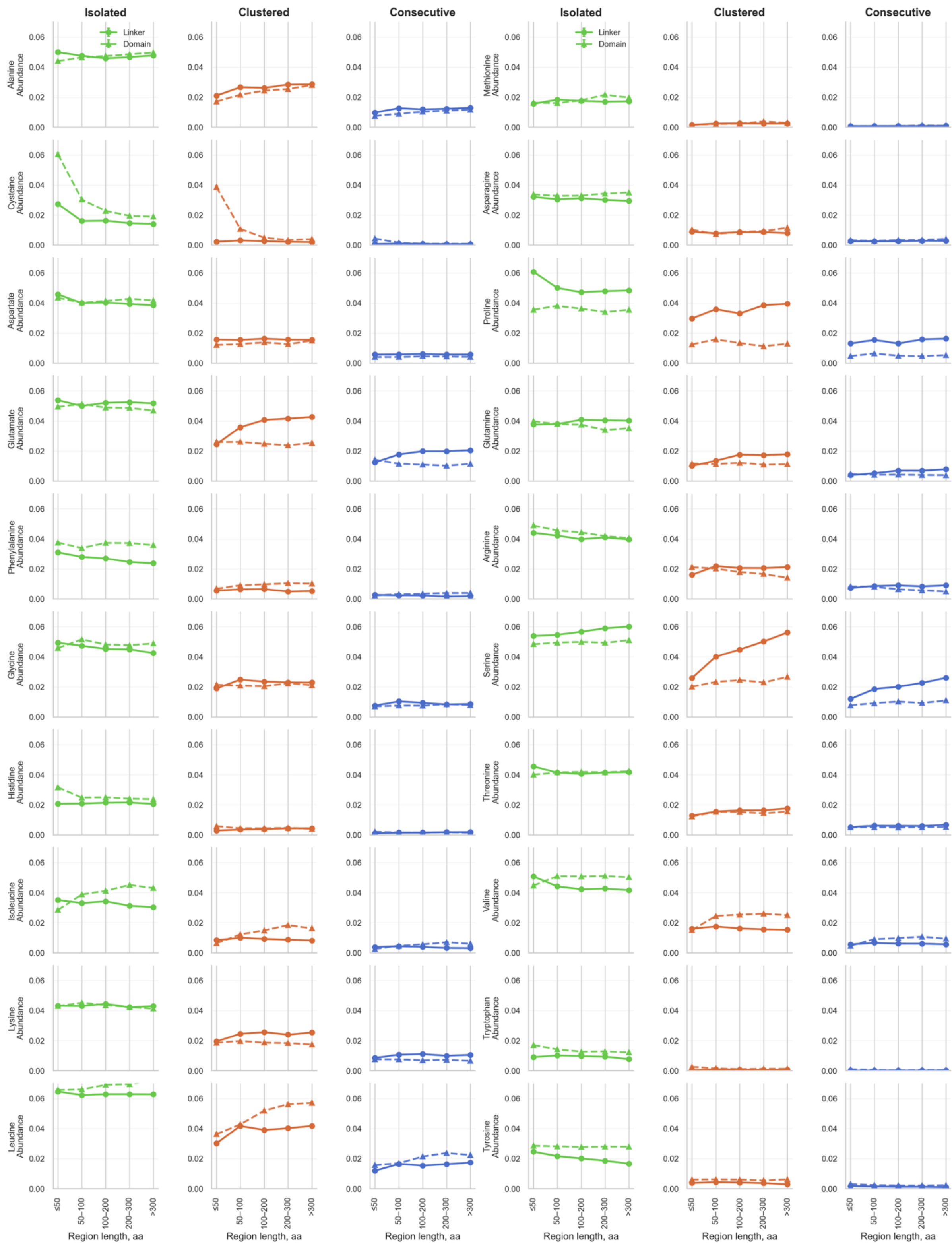

***Supplement 3. Linker and domain abundance patterns of 20 amino acids across region lengths*.**
