# Supplementary material for: Prolines in linkers of multi-domain proteins: Intrinsic regulators of misfolding and aggregation
Source: PLoS Comput Biol. 2026 Jul 31;22(7):e1014588. doi: 10.1371/journal.pcbi.1014588 (PMC13456264; doi:10.1371/journal.pcbi.1014588)
Supplement: Supplementary file 5 [file pcbi.1014588.s005.pdf]

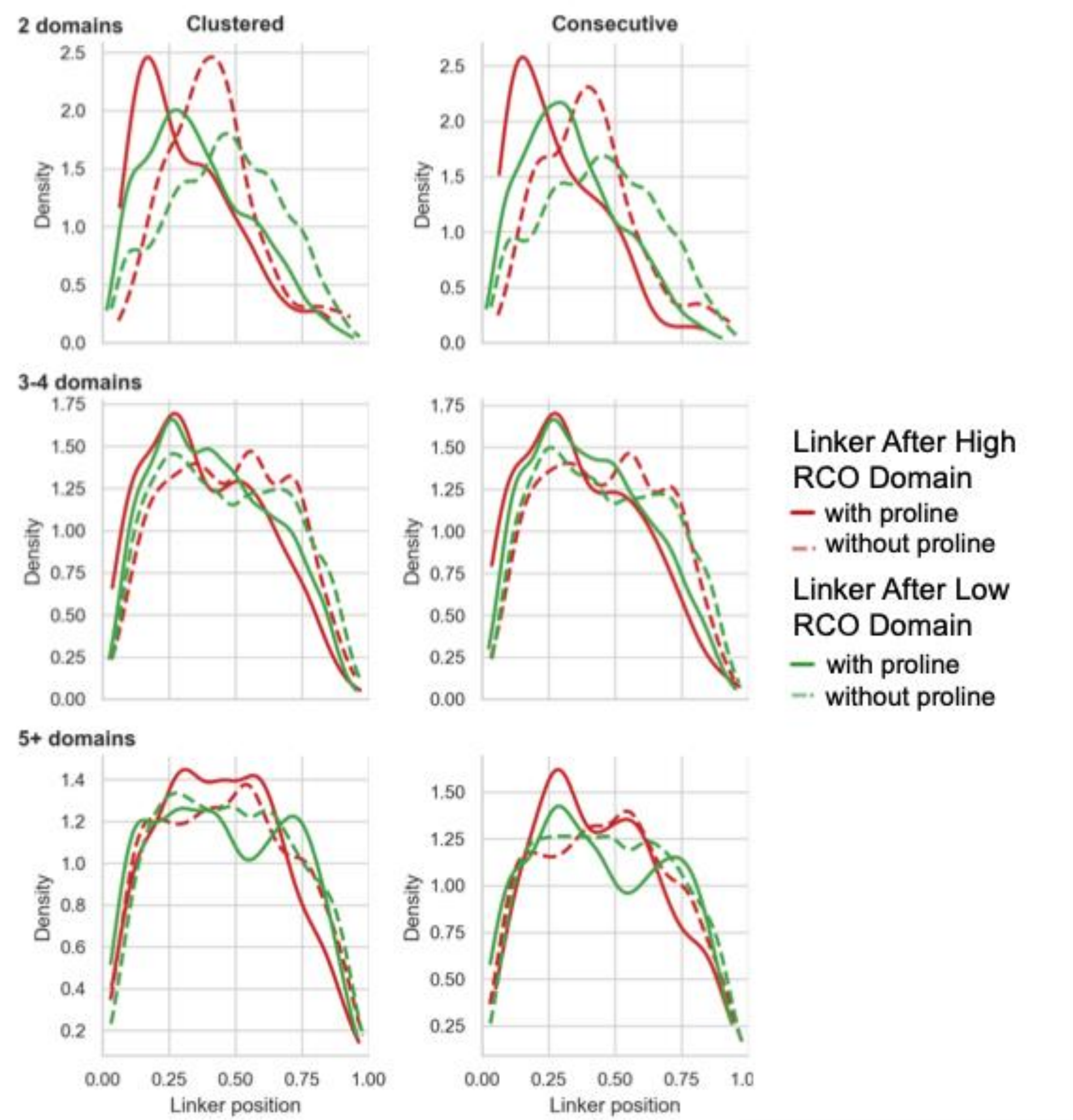

Supplement 5. Positional distribution of proline-containing linkers across protein domain-count groups.
