# Supplementary material for: Prolines in linkers of multi-domain proteins: Intrinsic regulators of misfolding and aggregation
Source: PLoS Comput Biol. 2026 Jul 31;22(7):e1014588. doi: 10.1371/journal.pcbi.1014588 (PMC13456264; doi:10.1371/journal.pcbi.1014588)

WEIZMANN INSTITUTE OF SCIENCE

Prof. Yaakov (Koby) Levy, Head
Dept. of Chemical & Structural Biology
Weizmann Institute of Science,
Rehovot, 76100, Israel

Phone: ++972-(0)8-934 4587
Fax: ++972-(0)8-934 4136

Koby.Levy@weizmann.ac.il

9 July 2026

Dear Editor,

Dear Prof. Jianhan Chen,

Thank you for handling our manuscript and for providing a thorough review. We appreciate the constructive comments and suggestions of the three reviewers. Following their comments we have revised the manuscript, and we believe the manuscript has been improved. The revision includes some new analysis and some modifications either in the figures (Figures 8) or the text. Following the reviewers' comments we also extended the SI documents by adding four additional figures (Figs. S2-S5).

Following is a detailed response to each of the reviewers' comments:

Reviewer #1

Method/analytical related part:

1) It would be helpful to clarify how overlapping domain annotations from HMMER/Pfam were merged. Specifically, were domains merged upon any overlap, or was a threshold applied? This choice may affect the identification of inter-domain linkers (≥ 3 residues), as small overlaps or boundary variations could eliminate or introduce short linker regions, particularly in repeat-containing or domain boundary-ambiguous cases.

We thank the reviewer for this comment. Overlapping domain hits were merged whenever their annotated residue ranges overlapped, we did not apply an additional overlap threshold. This was done to avoid introducing artificial short linker segments caused by minor boundary differences between neighboring hits. In addition, repeat-containing regions were treated separately: adjacent merged regions were further combined when both were annotated in Pfam as repeat domains and belonged to the

same repeat family, so that structurally interdependent tandem repeats were represented as extended repeat units. Linkers were then defined only between the resulting non-overlapping merged domain regions. A discussion was added on page 7.

2) Following the first question, it would be helpful to clarify how "non–proline-rich" regions were identified. Specifically, are these regions defined independently of whether proline residues are clustered or occur consecutively? Based on Figure 4A (dashed lines), it is not entirely clear why the positions of non–proline-rich linker regions appear identical between the clustered and consecutive proline cases.

   In this analysis (Fig. 4b), 'proline-rich' does not refer to a continuous measure of proline abundance. Rather, linker regions were classified in a binary way according to whether they contained at least one motif of the relevant type (clustered in general or consecutive). A discussion was added page 15 and the legend of Fig. 4 was revised accordingly.

3) I appreciate the Z-score analysis based on composition-preserving sequence shuffling, which is a sensible empirical framework for testing whether clustered proline abundance exceeds random expectation! At the same time, I wonder whether the authors could discuss how this approach compares with an explicit combinatorial treatment of proline patterning. For a linker of length L containing n_P prolines, the total number of composition-preserving arrangements is $\binom{L}{n_P}$, and the probability of a given organization class could in principle be written as $P(\text{pattern}) = \Omega_{\text{pattern}}/\binom{L}{n_P}$. For example, the probability of observing at least one consecutive proline pair is $1 - \binom{L-n_P+1}{n_P}/\binom{L}{n_P}$, whereas the probability that all prolines are isolated under the manuscript's definition is $\binom{L-3(n_P-1)}{n_P}/\binom{L}{n_P}$. This analytical might be useful when the authors note that Z-scores are difficult to interpret for short linkers.

   As already stated in the manuscript the Z-score approach is not informative for short linkers (<50 aa), because the constrained sequence space makes high clustered-proline abundance relatively likely by chance. Consequently, short linkers do not reach the significance threshold of 1.96, and we therefore interpret the Z-score analysis mainly for longer linkers.

4) How do the authors differ RCO high vs. low (like 0.15 they used)?

   We used RCO > 0.15 as a cutoff to define a high-RCO subset. In our dataset, this threshold lies above the 80th percentile of the empirical RCO distribution and therefore captures a relatively small upper-tail fraction of domains with comparatively high topological complexity. A discussion was added page 18.

5) The KDE analysis of linker positions may be influenced by inherent structural bias, as linkers are defined between domains and the first linker is necessarily located near the N-terminus. Proteins with fewer domains will therefore contribute disproportionately

to N-terminal linker positions. Therefore, it is unclear whether the reported enrichment reflects a true biological trend or an artifact of domain organization.

We thank the reviewer for this important point. We agree that linker-position KDEs can be influenced by multidomain architecture, particularly because proteins with fewer domains contribute disproportionately to early linker positions. To address this directly, we repeated the analysis after stratifying proteins by domain count (2 domains, 3–4 domains, and 5+ domains). This showed that the positional shift is strongest in proteins with fewer domains, but it remains detectable across domain-count groups, especially for linkers containing consecutive prolines. A discussion was added page 16 and supplement Fig. 5.

Biological-related questions:

1) Could the authors extend the analysis in Figure 2 to include all 20 amino acids? It would be interesting to assess whether other amino acid (i.e., Ala/Gln) exhibit similar clustering behavior in linker regions as observed for Proline. In particular, disordered proteins are known to contain poly(A)/poly(Q) stretches, and poly(Q) tracts are associated with aggregation.

   We thank the reviewer for this suggestion. To address this point, we extended the analysis to all 20 amino acids. This showed that some residues, particularly glutamate and serine, display broadly similar enrichment/clustering tendencies in linker regions at the global level. By contrast, glutamine did not show a comparable pattern in our dataset, despite the known association of poly(Q) tracts with low-complexity and aggregation-prone regions. Importantly, however, when we examined the relationship to the flanking domains, the asymmetry observed in our study, namely the stronger association with the upstream domain than with the downstream domain, was specific to proline. Thus, while certain other amino acids can show linker-enriched clustering behavior in a general sense, the domain-context dependence that motivated our interpretation appears to be most pronounced for proline. A discussion was added on page 14 and we added Fig. SI3 to the Supporting Information document.

2) Following the above point, the finding that proline clustering in linkers is more strongly associated with the upstream domain than the downstream domain is very interesting! I am wondering whether this effect is specific to proline, or whether other amino acid classes might exhibit similar positional or clustering patterns.

   Indeed, we followed the reviewer suggestion and examined the linkage between the clustering of each amino acid and the folding properties of the upstream and downstream domain. No residues showed similar behavior to proline. A discussion was dded page 15 and the new Fig. S4 in the Supporting Information.

3) How might intra-domain flexible or linker-like regions affect the CO/RCO analyses? Since CO/RCO is calculated from AlphaFold2-predicted structures, inclusion of such regions within annotated domains could influence contact-based metrics and bias estimates of folding complexity. In addition, AlphaFold2 is known to have reduced accuracy in disordered regions, which may further affect the reliability of these measurements. It would be helpful to clarify whether any filtering (like based on pLDDT) was applied, or to discuss the potential impact of including these regions.
   We thank the reviewer for this comment. We assessed this directly by calculating mean domain pLDDT values from the AlphaFold models used in the domain analysis. We then repeated the analysis using only domains with a mean pLDDT score above 70. A comment was added page 8.

4) The reported correlation between proline-rich linkers and aggregation-prone domains is an interesting observation. However, the mechanistic basis for this relationship is not entirely clear. Given that proline is generally considered a structure-disrupting residue and can inhibit β-sheet formation, it would be helpful if the authors could further discuss how proline enrichment in linkers might contribute to aggregation propensity, or whether this effect is indirect. In addition, the authors could consider plotting Figure 8B using density-based coloring to improve clarity as the data points are highly concentrated.
   Because proline is generally structure-disrupting and disfavors beta-sheet propagation, the association we observe is more consistent with a potential buffering or insulating role of proline-rich linkers near aggregation-prone domains than with a direct aggregation-promoting effect.

   We thank the reviewer for this comment. We agree that our aggregation-related analyses identify a statistical association rather than demonstrate a direct anti-aggregation mechanism. We have therefore revised the text throughout this section to avoid causal wording and to clarify that proline-rich or proline-clustered linkers are associated with aggregation-prone domains, but that this does not by itself prove that they suppress aggregation. We also illustrated the existence of clustered prolines for the linker that precedes the fibril-forming segment in tau protein. We have extended the discussion on the aggregation on page 23 as well as in the Discussion section. Following the reviewer's suggestions, we modified the colors of the density map in Fig. 8B changed

Reviewer #2:

1) There are many typos. These should be corrected.

We checked for typos and corrected them wherever necessary.

2) Page 8 indicates that "isolated" prolines are those that do not have another proline within two residues. Does this mean the a sequence PXP would not be considered "isolated"? As written, it seems like an arbitrary criterion that PXXP would be isolated, but PXP would not. Please clarify the rationale for this choice.

The defining characteristics of isolated, clustered, and consecutive prolines are described in the Methods and are also illustrated schematically in Fig. 1A. Under our definition, a PXP arrangement is not considered isolated, because another proline occurs within two residues, whereas PXXXP falls outside that range and is therefore classified as isolated.

3) page 12 indicates that residues are considered to be in contact if the CA atoms are within 6 Å. Why were CA distances used to identify contacts, rather than using atom-based identification? Also, 6Å seems very short for CA atoms. Wouldn't this basically limit the analysis to backbone-backbone contacts, which could potentially lead to significant changes in results. It is recommended that an atom-based contact measure be used, or perhaps just a longer CA-CA cutoff. The results may be robust. But, if they are sensitive to this choice, it would be important to understand why.

We thank the reviewer for this comment. We agree that the contact definition should be clarified and that testing robustness to this choice is important. In response, we repeated the analysis using an atom-based contact definition with an 8 Å cutoff and obtained the same overall outcome. We now note this explicitly in the manuscript and include the corresponding analysis in the Supplementary Information, showing that the main conclusions are not dependent on the original contact definition. This is discussed on page 14 and Fig S2 in the Supporting Information.

4) Page 13 states "to compare how disorder-promoting residues, such as proline and glycine...". Proline is thought to be more rigid, not more flexible, than a typical residue. With this in mind, it is not clear what is intended by the term "disorder-promoting"

We thank the reviewer for this question. We agree that proline should not be described as ‘more flexible’ in the same sense as glycine. Our intended meaning was that proline is associated with disorder-promoting sequence properties, because it disrupts regular secondary structure and is frequently enriched in disordered regions, despite being locally conformationally restricted. We have stated this explicitly in the Introduction.

5) page 14 states "the upstream domain i exerts the primary influence". I don't think "exerts" is appropriate at this point, as it would imply some form of causality, which I don't think is the intention of the authors.

Following the reviewer’s suggestion we changed to “upstream domain i shows the stronger association with proline clustering”

6) The final results section "Proline clustering emerges in aggregation contexts" is not as convincing as the preceding sections. Figure 8B is described as exhibiting a correlation, but the correlation is quite weak. Figures 8C and 8D also show very weak trends. It is not clear if these minor differences are meaningful, even if they are technically statistically distinct. Connecting these differences to biological impact would be helpful.

We thank the reviewer for this comment. We agree that our aggregation-related analyses identify a statistical association rather than demonstrate a direct anti-aggregation mechanism. We have therefore revised the text throughout this section to avoid causal wording and to clarify that proline-rich or proline-clustered linkers are associated with aggregation-prone domains, but that this does not by itself prove that they suppress aggregation. We also illustrated the existence of clustered prolines for the linker that precedes the fibril-forming segment in tau protein, aiming to have tighter connection to biological cases. We have extended the discussion on the aggregation on page 23 as well as in the Discussion section.

Reviewer #3

1. The main conclusion that proline clusters act as intrinsic regulators of co-translational folding and aggregation is not directly supported. The current data show correlations between proline clustering and domain/linker properties, but they do not establish a causal role. The title, abstract, and discussion should be toned down accordingly.

We thank the reviewer for this thoughtful comment. In response, we revised the Discussion and Abstract to tone down our conclusions and more clearly distinguish between the correlations observed in our study and their potential mechanistic implications. However, we prefer to retain the title, as we believe it accurately reflects the scope and main findings of the study without overstating causality.

2. The definition of "clustered prolines" is broader than experimentally established ribosome-stalling motifs. Prolines separated by up to two non-proline residues may not necessarily induce translational pausing. The authors should analyze specific motif classes separately, such as PP, PPP, XPPX, PXP, and PXXP motifs.

We agree that the definition of clustered prolines used here is broader than the set of experimentally established ribosome-stalling motifs. Accordingly, we analyze broad clustered prolines and consecutive prolines separately throughout the manuscript. We believe this distinction is sufficient for the present study, since the specific functional consequences of individual motif classes such as PP, PPP, XPPX, PXP, and PXXP are

not yet consistently defined in the literature. We therefore chose not to overinterpret individual motif subclasses, but instead to compare a broader clustering category with a narrower consecutive-proline category.

3. The aggregation analysis is interesting but does not demonstrate an anti-aggregation function. The authors show that linkers with high proline-clustering Z-scores tend to flank domains with higher predicted aggregation scores. However, this does not prove that the proline clusters suppress aggregation. Aggregation-prone domains may belong to specific structural or functional classes that also differ in RCO, domain length, β-content, or linker disorder. The authors should avoid concluding that proline clusters reduce aggregation unless additional validation is provided.
We thank the reviewer for this comment. We agree that our aggregation-related analyses identify a statistical association rather than demonstrate a direct anti-aggregation mechanism. We have therefore revised the text throughout this section to avoid causal wording and to clarify that proline-rich or proline-clustered linkers are associated with aggregation-prone domains, but that this does not by itself prove that they suppress aggregation. We also illustrated the existence of clustered prolines for the linker that precedes the fibril-forming segment in tau protein, aiming to have tighter connection to biological cases. We have extended the discussion on the aggregation on page 23 as well as in the Discussion section.

4. The Z-score analysis is problematic for short linkers, yet short linkers are central to the proposed mechanism. The authors state that Z-scores are not informative for short linkers, but they also argue that proline enrichment in short linkers downstream of high-RCO domains is especially important for providing translational pauses. A more appropriate null model for short linkers should be used.
As already noted in the manuscript, Z-scores are not informative for short linkers. Importantly, this analysis is used in our study for the aggregation-related question of whether clustered proline abundance exceeds random expectation, rather than as the basis for the inference about short proline-containing linkers downstream of high-RCO domains. That latter observation is based on motif occurrence and domain/linker context, not on Z-score significance in short linkers.

5. Additional negative controls are needed to demonstrate proline specificity. Glycine alone is not a sufficient control. The authors should analyze other residue clusters, such as serine/threonine-rich, charged, glutamine/asparagine-rich, or general low-complexity regions. This would help determine whether the observed patterns are specific to proline clustering or reflect general properties of disordered linkers.
We thank the reviewer for this suggestion. To address this point, we extended the analysis to all 20 amino acids. This showed that some residues, particularly glutamate

and serine, display broadly similar enrichment/clustering tendencies in linker regions at the global level. By contrast, glutamine did not show a comparable pattern in our dataset, despite the known association of poly(Q) tracts with low-complexity and aggregation-prone regions. Importantly, however, when we examined the relationship to the flanking domains, the asymmetry observed in our study, namely the stronger association with the upstream domain than with the downstream domain, was specific to proline. Thus, while certain other amino acids can show linker-enriched clustering behavior in a general sense, the domain-context dependence that motivated our interpretation appears to be most pronounced for proline. We examined the linkage between the clustering of each amino acid and the folding properties of the upstream and downstream domain. No residues showed similar behavior to proline. A discussion was added on pages 14, 15 and the new Figs. S3 and S4 in the Supporting Information.

6. A biologically relevant case study would greatly strengthen the manuscript. Tau is an obvious example because it contains a well-known proline-rich region adjacent to the aggregation-prone microtubule-binding repeat region. The authors could map isolated, clustered, and consecutive prolines along Tau, compare the proline-clustering profile with predicted aggregation propensity, and test whether the proline-rich region is positioned in a way consistent with their proposed anti-aggregation or spacer model.
We thank the reviewer for this suggestion. In response, we added a new analysis of the tau protein to Figure 8. This analysis demonstrates the presence of clustered prolines in the linker preceding the fibril-forming segment. The proline-clustering Z-score increases progressively across the 150-residue region upstream of the fibril-forming segment, reaching the significance threshold ($Z = 1.96$; see Fig. 1) near the fibril-forming segment (Fig. 8E).

Minor comments:

1. The authors should report effect sizes and confidence intervals, not only very small p values.
We incorporated confidence intervals into many of the presented analyses.

2. The threshold RCO > 0.15 should be justified, and the robustness of the conclusions to alternative thresholds should be tested.
We used RCO > 0.15 as a cutoff to define a high-RCO subset. In our dataset, this threshold lies above the 80th percentile of the empirical RCO distribution and therefore captures a relatively small upper-tail fraction of domains with comparatively high topological complexity. A discussion was added page 18.

3. AlphaFold-derived structures should be quality-controlled, for example by pLDDT filtering, before calculating contact order.

We thank the reviewer for this comment. We assessed this directly by calculating mean domain pLDDT values from the AlphaFold models used in the domain analysis. We then repeated the analysis using only domains with a mean pLDDT score above 70. A comment was added page 8.

4. The Pfam-based linker definition may misclassify unannotated structured regions, coiled coils, repeat regions, or functional low-complexity domains as linkers. This limitation should be discussed.

We agree that defining linkers from Pfam annotations has inherent limitations. We now discuss this limitation explicitly in the revised manuscript and note that such cases could introduce some uncertainty in linker assignment. A comment was added on page 7.

Yaakov (Koby) Levy

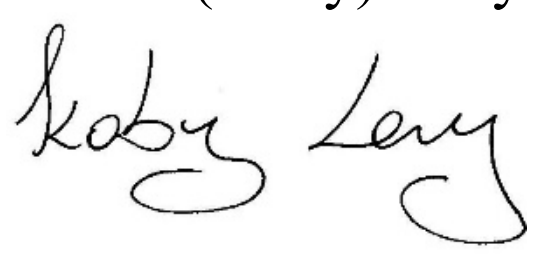

Supplement: Supplementary file 7 [file pcbi.1014588.s009.pdf]
