# Supplementary material for: Prolines in linkers of multi-domain proteins: Intrinsic regulators of misfolding and aggregation
Source: PLoS Comput Biol. 2026 Jul 31;22(7):e1014588. doi: 10.1371/journal.pcbi.1014588 (PMC13456264; doi:10.1371/journal.pcbi.1014588)
Supplement: Supplementary file 8 [file pcbi.1014588.s007.docx]

Simona *et al*. present a proteome-wide analysis of how proline organization within inter-domain linkers of multi-domain proteins relates to folding, aggregation, and stability. This timely study bridges an important gap by linking proline patterning, beyond overall abundance, to potential biological functions. In particular, the authors suggest that clustered and consecutive prolines may modulate co-translational folding through sequence-encoded translational pauses, and are associated with reduced aggregation propensity and increased protein half-life. By systematically analyzing proline distribution, structural complexity, and evolutionary conservation, the work provides new insights into how sequence organization in disordered regions contributes to proteostasis. Overall, the manuscript is biologically informative and offers an interesting perspective on the functional role of proline patterning in multidomain proteins. The manuscript would benefit from addressing the following points:

Method/analytical related part:

1. It would be helpful to clarify how overlapping domain annotations from HMMER/Pfam were merged. Specifically, were domains merged upon any overlap, or was a threshold applied? This choice may affect the identification of inter-domain linkers (≥ 3 residues), as small overlaps or boundary variations could eliminate or introduce short linker regions, particularly in repeat-containing or domain boundary-ambiguous cases.
2. Following the first question, it would be helpful to clarify how “non–proline-rich” regions were identified. Specifically, are these regions defined independently of whether proline residues are clustered or occur consecutively? Based on Figure 4A (dashed lines), it is not entirely clear why the positions of non–proline-rich linker regions appear identical between the clustered and consecutive proline cases.
3. I appreciate the Z-score analysis based on composition-preserving sequence shuffling, which is a sensible empirical framework for testing whether clustered proline abundance exceeds random expectation! At the same time, I wonder whether the authors could discuss how this approach compares with an explicit combinatorial treatment of proline patterning. For a linker of length $L$ containing $n_{P}$ prolines, the total number of composition-preserving arrangements is $\binom{L}{n_{P}}$, and the probability of a given organization class could in principle be written as $P(\text{pattern})=\Omega_{\text{pattern}}/\binom{L}{n_{P}}$. For example, the probability of observing at least one consecutive proline pair is $1-\binom{L-n_{P}+1}{n_{P}}/\binom{L}{n_{P}}$, whereas the probability that all prolines are isolated under the manuscript’s definition is $\binom{L-3(n_{P}-1)}{n_{P}}/\binom{L}{n_{P}}$. This analytical might be useful when the authors note that Z-scores are difficult to interpret for short linkers.
4. How do the authors differ RCO high vs. low (like 0.15 they used)?
5. The KDE analysis of linker positions may be influenced by inherent structural bias, as linkers are defined between domains and the first linker is necessarily located near the N-terminus. Proteins with fewer domains will therefore contribute disproportionately to N-terminal linker positions. Therefore, it is unclear whether the reported enrichment reflects a true biological trend or an artifact of domain organization.

Biological-related questions:

1. Could the authors extend the analysis in Figure 2 to include all 20 amino acids? It would be interesting to assess whether other amino acid (i.e., Ala/Gln) exhibit similar clustering behavior in linker regions as observed for Proline. In particular, disordered proteins are known to contain poly(A)/poly(Q) stretches, and poly(Q) tracts are associated with aggregation.
2. Following the above point, the finding that proline clustering in linkers is more strongly associated with the upstream domain than the downstream domain is very interesting! I am wondering whether this effect is specific to proline, or whether other amino acid classes might exhibit similar positional or clustering patterns.
3. How might intra-domain flexible or linker-like regions affect the CO/RCO analyses? Since CO/RCO is calculated from AlphaFold2-predicted structures, inclusion of such regions within annotated domains could influence contact-based metrics and bias estimates of folding complexity. In addition, AlphaFold2 is known to have reduced accuracy in disordered regions, which may further affect the reliability of these measurements. It would be helpful to clarify whether any filtering (like based on pLDDT) was applied, or to discuss the potential impact of including these regions.
4. The reported correlation between proline-rich linkers and aggregation-prone domains is an interesting observation. However, the mechanistic basis for this relationship is not entirely clear. Given that proline is generally considered a structure-disrupting residue and can inhibit β-sheet formation, it would be helpful if the authors could further discuss how proline enrichment in linkers might contribute to aggregation propensity, or whether this effect is indirect. In addition, the authors could consider plotting Figure 8B using density-based coloring to improve clarity as the data points are highly concentrated.
