# Supplementary material for: Prolines in linkers of multi-domain proteins: Intrinsic regulators of misfolding and aggregation
Source: PLoS Comput Biol. 2026 Jul 31;22(7):e1014588. doi: 10.1371/journal.pcbi.1014588 (PMC13456264; doi:10.1371/journal.pcbi.1014588)
Supplement: Peer review history

# Peer review: Decision Letter 0

**DOI:** 10.1371/journal.pcbi.1014588.r001

**Authors:** Nir Ben-Tal, Jianhan Chen

3 May 2026

PCOMPBIOL-D-26-00725

Prolines in Linkers of Multi-Domain Proteins: Intrinsic Regulators of Misfolding and Aggregation

PLOS Computational Biology

Dear Dr. Levy,

Thank you for submitting your manuscript to PLOS Computational Biology. After careful consideration, we feel that it has merit but does not fully meet PLOS Computational Biology's publication criteria as it currently stands. Therefore, we invite you to submit a revised version of the manuscript that addresses the points raised during the review process.

Please submit your revised manuscript by Jul 03 2026 11:59PM. If you will need more time than this to complete your revisions, please reply to this message or contact the journal office at ploscompbiol@plos.org. When you're ready to submit your revision, log on to https://www.editorialmanager.com/pcompbiol/ and select the 'Submissions Needing Revision' folder to locate your manuscript file.

Please include the following items when submitting your revised manuscript:

* A letter that responds to each point raised by the editor and reviewer(s). You should upload this letter as a separate file labeled 'Response to Reviewers'. This file does not need to include responses to formatting updates and technical items listed in the 'Journal Requirements' section below.

* A marked-up copy of your manuscript that highlights changes made to the original version. You should upload this as a separate file labeled 'Revised Manuscript with Track Changes'.

* An unmarked version of your revised paper without tracked changes. You should upload this as a separate file labeled 'Manuscript'.

If you would like to make changes to your financial disclosure, competing interests statement, or data availability statement, please make these updates within the submission form at the time of resubmission. Guidelines for resubmitting your figure files are available below the reviewer comments at the end of this letter.

We look forward to receiving your revised manuscript.

Kind regards,

Jianhan Chen, Ph.D.

Academic Editor

PLOS Computational Biology

Nir Ben-Tal

Section Editor

PLOS Computational Biology

**Additional Editor Comments:**

The manuscript is well received by the reviewers, with some modest and minor concerns raised. I will greatly appreciate a revised manuscript with all points satisfactorily addressed.

**Journal Requirements:**

If the reviewer comments include a recommendation to cite specific previously published works, please review and evaluate these publications to determine whether they are relevant and should be cited. There is no requirement to cite these works unless the editor has indicated otherwise.

1) Please ensure that the CRediT author contributions listed for every co-author are completed accurately and in full.

At this stage, the following Authors/Authors require contributions: Simona Manasra, and Yaakov Koby Levy. Please ensure that the full contributions of each author are acknowledged in the "Add/Edit/Remove Authors" section of our submission form.

The list of CRediT author contributions may be found here: https://journals.plos.org/ploscompbiol/s/authorship#loc-author-contributions

2) We ask that a manuscript source file is provided at Revision. Please upload your manuscript file as a .doc, .docx, .rtf or .tex. If you are providing a .tex file, please upload it under the item type u2018LaTeX Source Fileu2019 and leave your .pdf version as the item type u2018Manuscriptu2019.

3) Your manuscript's sections are not in the correct order.  Please amend to the following order: Abstract, Introduction, Results, Discussion, and Methods

4) Please upload all main figures as separate Figure files in .tif or .eps format. For more information about how to convert and format your figure files please see our guidelines:

https://journals.plos.org/ploscompbiol/s/figures

5) We note that your Data Availability Statement is currently as follows: "All relevant data are within the manuscript and its Supporting Information files.". Please confirm at this time whether or not your submission contains all raw data required to replicate the results of your study. Authors must share the “minimal data set” for their submission. PLOS defines the minimal data set to consist of the data required to replicate all study findings reported in the article, as well as related metadata and methods (https://journals.plos.org/plosone/s/data-availability#loc-minimal-data-set-definition).

For example, authors should submit the following data:

1) The values behind the means, standard deviations and other measures reported;

2) The values used to build graphs;

3) The points extracted from images for analysis..

Authors do not need to submit their entire data set if only a portion of the data was used in the reported study.

If your submission does not contain these data, please either upload them as Supporting Information files or deposit them to a stable, public repository and provide us with the relevant URLs, DOIs, or accession numbers. For a list of recommended repositories, please see https://journals.plos.org/plosone/s/recommended-repositories.

If there are ethical or legal restrictions on sharing a de-identified data set, please explain them in detail (e.g., data contain potentially sensitive information, data are owned by a third-party organization, etc.) and who has imposed them (e.g., an ethics committee). Please also provide contact information for a data access committee, ethics committee, or other institutional body to which data requests may be sent. If data are owned by a third party, please indicate how others may request data access.

**Reviewers' comments:**

Reviewer's Responses to Questions

**Comments to the Authors:**

**Please note here if the review is uploaded as an attachment.**

Reviewer #1: Attached

Reviewer #2: This study looks at the distribution of proline clusters in protein linkers. Prolines are unusual, since they have an atypical side chain that restricts mobility. In the current study, a bioinformatic analysis is applied to identify if there are preferences for proline stretches, which may be linked to properties of co-translational folding. The main finding is that proline stretches tend to be positioned in linkers that follow domains that have complex folds. These complex folds are likely to be slow-forming, which can make them prone to aggregation. By encoding polyproline segments after these domains, the protein sequence essentially encodes a pause in translation, which can allow for folding to occur before translating subsequent domains. I have some minor comments and questions that should be addressed prior to publication.

1) There are many typos. These should be corrected.

2) Page 8 indicates that "isolated" prolines are those that do not have another proline within two residues. Does this mean the a sequence PXP would not be considered "isolated"? As written, it seems like an arbitrary criterion that PXXP would be isolated, but PXP would not. Please clarify the rationale for this choice.

3) page 12 indicates that residues are considered to be in contact if the CA atoms are within 6 Å. Why were CA distances used to identify contacts, rather than using atom-based identification? Also, 6Å seems very short for CA atoms. Wouldn't this basically limit the analysis to backbone-backbone contacts, which could potentially lead to significant changes in results. It is recommended that an atom-based contact measure be used, or perhaps just a longer CA-CA cutoff. The results may be robust. But, if they are sensitive to this choice, it would be important to understand why.

4 ) Page 13 states "to compare how disorder-promoting residues, such as proline and glycine...". Proline is thought to be more rigid, not more flexible, than a typical residue. With this in mind, it is not clear what is intended by the term "disorder-promoting"

5) page 14 states "the upstream domain i exerts the primary influence". I don't think "exerts" is appropriate at this point, as it would imply some form of causality, which I don't think is the intention of the authors.

6) The final results section "Proline clustering emerges in aggregation contexts" is not as convincing as the preceding sections. Figure 8B is described as exhibiting a correlation, but the correlation is quite weak. Figures 8C and 8D also show very weak trends. It is not clear if these minor differences are meaningful, even if they are technically statistically distinct. Connecting these differences to biological impact would be helpful.

Reviewer #3: In this manuscript, Manasra and Levy performed a proteome-wide analysis of proline organization in inter-domain linkers of multi-domain proteins. They report that clustered and consecutive prolines are enriched in linkers, particularly downstream of domains with high relative contact order, and suggest that such proline clusters may serve as intrinsic sequence-encoded pauses that facilitate co-translational folding and reduce misfolding or aggregation. The authors further show that linkers with enriched proline clusters are often adjacent to aggregation-prone domains and exhibit certain evolutionary conservation patterns. Overall, the manuscript is well written, and presents an interesting hypothesis and several potentially valuable observations, but the current evidence remains largely correlative. Several major issues should be addressed.

1. The main conclusion that proline clusters act as intrinsic regulators of co-translational folding and aggregation is not directly supported. The current data show correlations between proline clustering and domain/linker properties, but they do not establish a causal role. The title, abstract, and discussion should be toned down accordingly.

2. The definition of “clustered prolines” is broader than experimentally established ribosome-stalling motifs. Prolines separated by up to two non-proline residues may not necessarily induce translational pausing. The authors should analyze specific motif classes separately, such as PP, PPP, XPPX, PXP, and PXXP motifs.

3. The aggregation analysis is interesting but does not demonstrate an anti-aggregation function. The authors show that linkers with high proline-clustering Z-scores tend to flank domains with higher predicted aggregation scores. However, this does not prove that the proline clusters suppress aggregation. Aggregation-prone domains may belong to specific structural or functional classes that also differ in RCO, domain length, β-content, or linker disorder. The authors should avoid concluding that proline clusters reduce aggregation unless additional validation is provided.

4. The Z-score analysis is problematic for short linkers, yet short linkers are central to the proposed mechanism. The authors state that Z-scores are not informative for short linkers, but they also argue that proline enrichment in short linkers downstream of high-RCO domains is especially important for providing translational pauses. A more appropriate null model for short linkers should be used.

5. Additional negative controls are needed to demonstrate proline specificity. Glycine alone is not a sufficient control. The authors should analyze other residue clusters, such as serine/threonine-rich, charged, glutamine/asparagine-rich, or general low-complexity regions. This would help determine whether the observed patterns are specific to proline clustering or reflect general properties of disordered linkers.

6. A biologically relevant case study would greatly strengthen the manuscript. Tau is an obvious example because it contains a well-known proline-rich region adjacent to the aggregation-prone microtubule-binding repeat region. The authors could map isolated, clustered, and consecutive prolines along Tau, compare the proline-clustering profile with predicted aggregation propensity, and test whether the proline-rich region is positioned in a way consistent with their proposed anti-aggregation or spacer model.

Minor comments:

1. The authors should report effect sizes and confidence intervals, not only very small p values.

2. The threshold RCO > 0.15 should be justified, and the robustness of the conclusions to alternative thresholds should be tested.

3. AlphaFold-derived structures should be quality-controlled, for example by pLDDT filtering, before calculating contact order.

4. The Pfam-based linker definition may misclassify unannotated structured regions, coiled coils, repeat regions, or functional low-complexity domains as linkers. This limitation should be discussed.

**********

**Have the authors made all data and (if applicable) computational code underlying the findings in their manuscript fully available?**

The PLOS Data policy requires authors to make all data and code underlying the findings described in their manuscript fully available without restriction, with rare exception (please refer to the Data Availability Statement in the manuscript PDF file). The data and code should be provided as part of the manuscript or its supporting information, or deposited to a public repository. For example, in addition to summary statistics, the data points behind means, medians and variance measures should be available. If there are restrictions on publicly sharing data or code —e.g. participant privacy or use of data from a third party—those must be specified.

Reviewer #1: Yes

Reviewer #2: None

Reviewer #3: None

**********

PLOS authors have the option to publish the peer review history of their article (what does this mean?). If published, this will include your full peer review and any attached files.

If you choose “no”, your identity will remain anonymous but your review may still be made public.

**Do you want your identity to be public for this peer review?**  For information about this choice, including consent withdrawal, please see our Privacy Policy.

Reviewer #1: No

Reviewer #2: No

Reviewer #3: No

[NOTE: If reviewer comments were submitted as an attachment file, they will be attached to this email and accessible via the submission site. Please log into your account, locate the manuscript record, and check for the action link "View Attachments". If this link does not appear, there are no attachment files.]

**Figure resubmission:**

After uploading your figures to PLOS’s NAAS tool - https://ngplosjournals.pagemajik.ai/artanalysis, NAAS will process the files provided and display the results in the "Uploaded Files" section of the page as the processing is complete. If the uploaded figures meet our requirements (or NAAS is able to fix the files to meet our requirements), the figure will be marked as "fixed" above. If NAAS is unable to fix the files, a red "failed" label will appear above. When NAAS has confirmed that the figure files meet our requirements, please download the file via the download option, and include these NAAS processed figure files when submitting your revised manuscript.

**Reproducibility:**

To enhance the reproducibility of your results, we recommend that authors of applicable studies deposit laboratory protocols in protocols.io, where a protocol can be assigned its own identifier (DOI) such that it can be cited independently in the future. Additionally, PLOS ONE offers an option to publish peer-reviewed clinical study protocols. Read more information on sharing protocols at https://plos.org/protocols?utm_medium=editorial-email&utm_source=authorletters&utm_campaign=protocols

Attachment Submitted filename: review.docx

# Author comment: Author response to Decision Letter 1

**DOI:** 10.1371/journal.pcbi.1014588.r002

11 Jul 2026

Attachment Submitted filename: review_response_KL_2.pdf

# Editor report: Decision Letter 1

**DOI:** 10.1371/journal.pcbi.1014588.r003

**Authors:** Nir Ben-Tal, Jianhan Chen

16 Jul 2026

Dear Dr. Levy,

We are pleased to inform you that your manuscript 'Prolines in Linkers of Multi-Domain Proteins: Intrinsic Regulators of Misfolding and Aggregation' has been provisionally accepted for publication in PLOS Computational Biology.

Before your manuscript can be formally accepted you will need to complete some formatting changes, which you will receive in a follow up email. A member of our team will be in touch with a set of requests.

Please note that your manuscript will not be scheduled for publication until you have made the required changes, so a swift response is appreciated.

IMPORTANT: The editorial review process is now complete. PLOS will only permit corrections to spelling, formatting or significant scientific errors from this point onwards. Requests for major changes, or any which affect the scientific understanding of your work, will cause delays to the publication date of your manuscript.

Should you, your institution's press office or the journal office choose to press release your paper, you will automatically be opted out of early publication. We ask that you notify us now if you or your institution is planning to press release the article. All press must be co-ordinated with PLOS.

Thank you again for supporting Open Access publishing; we are looking forward to publishing your work in PLOS Computational Biology.

Best regards,

Jianhan Chen, Ph.D.

Academic Editor

PLOS Computational Biology

Nir Ben-Tal

Section Editor

PLOS Computational Biology

***********************************************************

# Editor report: Acceptance letter

**DOI:** 10.1371/journal.pcbi.1014588.r004

**Authors:** Nir Ben-Tal, Jianhan Chen

PCOMPBIOL-D-26-00725R1

Prolines in Linkers of Multi-Domain Proteins: Intrinsic Regulators of Misfolding and Aggregation

Dear Dr Levy,

I am pleased to inform you that your manuscript has been formally accepted for publication in PLOS Computational Biology. Your manuscript is now with our production department and you will be notified of the publication date in due course.

The corresponding author will soon be receiving a typeset proof for review, to ensure errors have not been introduced during production. Please review the PDF proof of your manuscript carefully, as this is the last chance to correct any errors. Please note that major changes, or those which affect the scientific understanding of the work, will likely cause delays to the publication date of your manuscript.

Soon after your final files are uploaded, unless you have opted out, the early version of your manuscript will be published online. The date of the early version will be your article's publication date. The final article will be published to the same URL, and all versions of the paper will be accessible to readers.

For Research, Software, and Methods articles, you will receive an invoice from PLOS for your publication fee after your manuscript has reached the completed accept phase. If you receive an email requesting payment before acceptance or for any other service, this may be a phishing scheme. Learn how to identify phishing emails and protect your accounts at https://explore.plos.org/phishing.

Thank you again for supporting PLOS Computational Biology and open-access publishing. We are looking forward to publishing your work!

With kind regards,

Sharmila Kamatchi

PLOS Computational Biology | Carlyle House, Carlyle Road, Cambridge CB4 3DN | United Kingdom ploscompbiol@plos.org | Phone +44 (0) 1223-442824 | ploscompbiol.org | @PLOSCompBiol
